# An Efficient One-Pot Green Protocol for the Synthesis of 5-Unsubstituted 3,4-Dihydropyrimidin-2(1*H*)-Ones Using Recyclable Amberlyst 15 DRY as a Heterogeneous Catalyst via Three-Component Biginelli-Like Reaction

**DOI:** 10.5402/2012/480989

**Published:** 2012-10-14

**Authors:** Srinivasa Rao Jetti, Divya Verma, Shubha Jain

**Affiliations:** Laboratory of Heterocycles, School of Studies in Chemistry & Biochemistry, Vikram University, Ujjain 456010, Madhya Pradesh, India

## Abstract

An environmentally benign green protocol for the synthesis of 5-unsubstituted 3,4-dihydropyrimidin-2(1*H*)-ones using Amberlyst 15 DRY as a recyclable catalyst has been developed. The use of resinous, nontoxic, thermally stable, and inexpensive Amberlyst 15 DRY, as a recyclable heterogeneous catalyst, makes the process simple with negligible chemical waste. Among the various solid acid catalysts Amberlyst 15 DRY was found to be the most efficient catalyst with regard to reaction time, yield, and ease of work-up procedure.

## 1. Introduction

Replacement of conventional, toxic, and polluting Bronsted and Lewis acid catalysts with ecofriendly reusable solid acid heterogeneous catalysts like acidic zeolites, clays, sulfated zirconia, and ion exchange resins is an area of current interest [1, 2]. The use of solid acid catalyst instead of liquids includes many advantages, such as reduced equipment corrosion, ease of product separation, recycling of the catalyst, and environmental acceptability. In the recent past ion exchange resins in general and styrene-DVB matrix resin sulfonic acid (Amberlyst 15 DRY) in particular, which are strongly acidic and chemically as well as thermally stable, have been found to be excellent catalysts for a variety of the major organic reactions like esterification, alkylation, acylation, and condensation [3–8].

**Figure 1:**
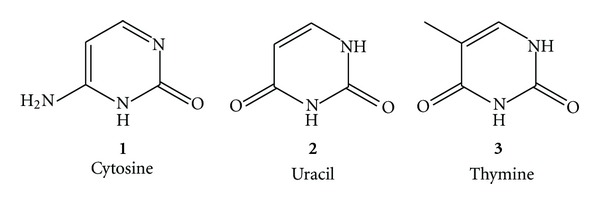


Pyrimidinones or dihydropyrimidinones (DHPMs) are well known for their wide range of bioactivities. Their applications in the field of drug research have stimulated the development of a wide range of synthetic methods for their preparation and chemical transformations. Out of the five major bases in nucleic acids three are pyrimidine derivatives which comprise of cytosine (**1**) which is found in DNA and RNA, uracil (**2**) in RNA and thymine, and (**3**) in DNA. Because of their involvement as bases in DNA and RNA, they have become very important in the world of synthetic organic chemistry. Aryl-substituted 3,4-dihydropyrimidin-2(1*H*)-one and their derivatives are an important class of substances in organic and medicinal chemistry (see Figure 1).

**Figure 2:**
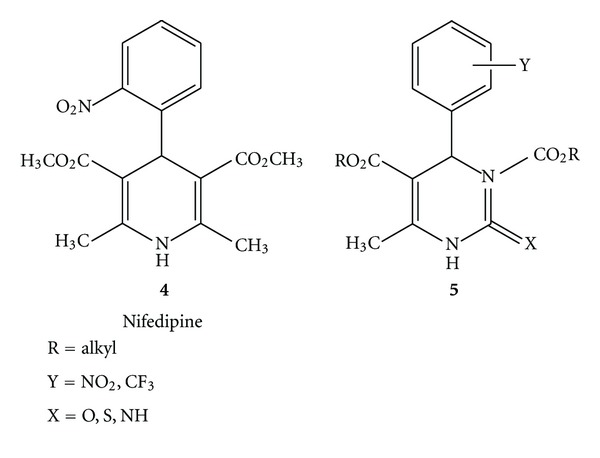


4-Aryl-1,4-dihydropyridines (DHPMs) of the Nifedipine type (**4**) [9] were first introduced into clinical medicine in 1975 and are still the most potent group of calcium channel modulators available for the treatment of cardiovascular diseases [10]. Dihydropyrimidines of type (**5**) show a very similar pharmacological profile, and in recent years, several related compounds were developed (**5**) that are equal in potency and duration of antihypertensive activity to classical and second-generation dihydropyridinedrugs [11]. (see Figure 2).

In an attempt to prepare DHPMs, different types of acidic catalysts such as H_2_SO_4_ [12], BF_3_·EtOH/CuCl [13], LaCl_3_·7H_2_O with catalytic concentrated HCl [14], CeCl_3_·7H_2_O [15], InCl_3_ [16], heteropolyacids [17], BiCl_3_ [18], Cu(OTf)_2_ [19], TMSCl [20], LiClO_4_ [21], LiBr [22], InBr_3_ [23], phenyl pyruvic acid [24], FeCl_3_·6H_2_O/HCl [25], TMSI [26], CdCl_2_ [27], CuCl_2_·2H_2_O–HCl [28], and ZnBr_2_ [29] have been used. However, inspite of their potential utility many of the existing methods suffer from the drawbacks, such as the use of strong acidic conditions, longer reaction times, tedious workup, environmental disposal problems, and lower yields of the products, leaving scope for further development of an efficient and versatile method for Biginelli reaction.

**Scheme 1:**
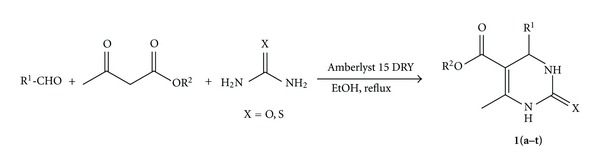


Growing concern about environmental damage leads to an urgent requirement for the development of ecofriendly technology and economic processes. It is of great practical importance to synthesize DHPM derivatives by the Biginelli reaction by using a solid acid catalyst, because of the ability to modify the acid strength, ease of handling, recycling of the catalyst, and environmental compatibility. In view of the above requirement, and as a part of our program towards green synthesis, we herein report a single-step and ecofriendly protocol for the synthesis of DHPM derivatives by the multicomponent reactions of 1,3-dicarbonyl compound, aldehydes, and urea (Scheme 1) over Amberlyst 15 DRY with good yields and selectivity.

## 2. Results and Discussion

To evaluate the catalytic effect of various ion exchange resins we started with the model reaction of ethylacetoacetate (1.0 mmol) with benzaldehyde (1.0 mmol) and urea (1.2 mmol) in refluxing ethanol without and with use of Amberlyst-70 and Amberlyst 15 DRY as catalysts to afford dihydropyrimidine **1a** and the results obtained were compared with those already reported using other catalysts [30, 31] (Table 1). It can be seen from Table 1 that Amberlyst 15 DRY is the most efficient among the five solid acidic ion exchange resins. It was found that 50 mg of Amberlyst 15 DRY is sufficient to carry out the Biginelli reaction successfully. An increase in the amount of Amberlyst 15 DRY to more than 50 mg showed no substantial improvement in the yield, whereas the yield is reduced by decreasing the amount of Amberlyst 15 DRY.

The effect of solvent on the reaction was studied (Table 2, entries 1–6) and ethanol was found to be the best solvent when considering the reaction yields and environmental damage.

The method can be used for wide range of reactants with different functional group. We have synthesized some novel compounds containing quinoline, pyrimidine, indole, and coumarin units (Table 3). All reactions proceeded expeditiously and delivered good yields with broad range of structurally diverse aromatic and heterocyclic aldehydes used in this condensation. *α*, *β*-Unsaturated aldehydes react selectively with aldehyde functional group, whereas acid sensitive heterocyclic aldehydes exclusively gave dihydropyrimidinones in high yield. We found that electron donating or withdrawing group on aromatic aldehydes gave almost good to excellent yield. In all the cases the pure product was isolated by simple filtration without use of any chromatography or cumbersome reaction workup.

**Figure 3:**
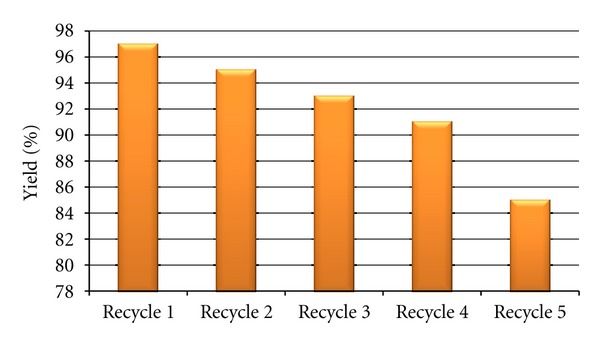
Graphical representation of recycle data for the reaction of EAA, benzaldehyde, and urea using Amberlyst 15 DRY.

The resin catalyst was separated from the reaction mixture by filtration and can be reused several times without any appreciable loss in activity, which clearly proves the recyclability and reusability of the catalyst (Figure 3). It is noteworthy to mention that these reactions are working well without using any phase transfer catalyst. Furthermore, the protocol has its advantages lying in the ease of separation of catalyst and the product, which can be achieved by simple filtration.

**Scheme 2:**
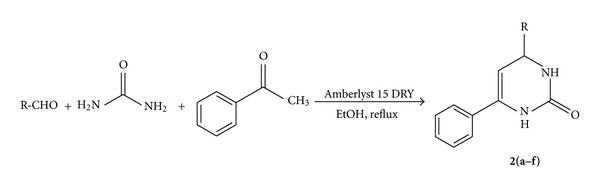


The formation of product **2** (Scheme 2) probably involves the activation of the carbonyl function by Amberlyst 15 DRY, thereby making the methyl group readily enolisable, which in turn reacts with aldehyde and urea-derived imine in a Michael-type step to produce **2** (Table 4).

**Scheme 3:**
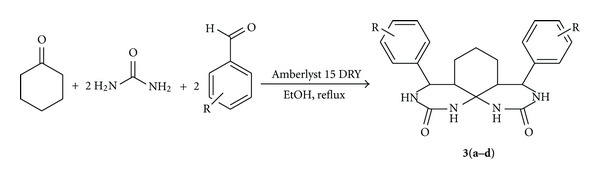


This investigation has been extended to cyclic ketones like cyclohexanone (Scheme 3). The products formed (**3a–d**) are listed in Table 5.

## 3. Experimental

### 3.1. General

**Figure 4:**
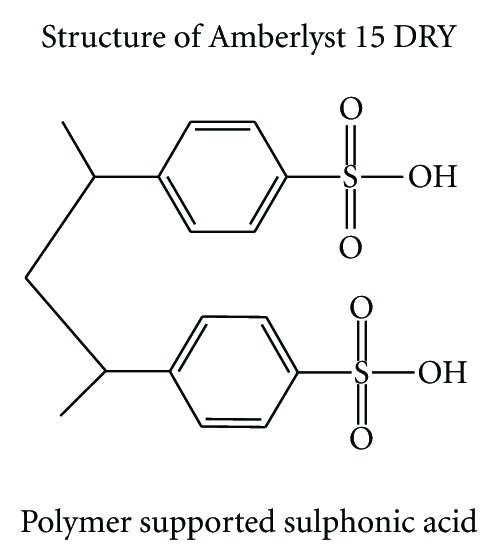


All solvents and reagents were purchased from Aldrich and Merck with high-grade quality and used without any purification. The Indion-130 and Indion-190 were purchased from Ion Exchange India Ltd. Nafion-H, Amberlyst-70, and Amberlyst 15 DRY were purchased from Aldrich. Melting points were determined on electrothermal apparatus by using open capillaries and are uncorrected. Thin-layer chromatography was accomplished on 0.2-mm precoated plates of silica gel G60 F254 (Merck). Visualization was made with UV light (254 and 365 nm) or with an iodine vapor. IR spectra were recorded on a FTIR-8400 spectrophotometer using DRS prob. ^1^H-NMR and ^13^C-NMR spectra were recorded in DMSO-*d*_6_ solutions on a Bruker AVANCE 400NMR spectrometer operating at 400 (^1^H) and 100 (^13^C) MHz. LCMS analysis (EI, 70 V) was performed on a Hewlett-Packard HP 5971 instrument. All compounds were characterized by comparison of physical and spectral data with reported data [32–35] (see Figure 4, Table 6).

### 3.2. General Procedure for the Synthesis of 4-Aryl Substituted 3,4-Dihydropyrimidin-2-(1H)-ones/thiones

A mixture of *β*-diketone (1.0 mmol), aldehyde (1.0 mmol), urea/thiourea (1.2 mmol), and Amberlyst 15 DRY (50 mg) in anhydrous ethanol (10 mL) was refluxed for an appropriate time as indicated by TLC. After completion of the reaction the catalyst was filtered and washed with ethyl acetate until free from organic material. The solvent was evaporated at reduced pressure and obtained solid was crystallized from ethanol to afford pure 3,4-dihydropyrimidin-2-(1*H*)-ones/thiones **1(a–t)** in excellent yields.

### 3.3. General Procedure for the Synthesis of 3,4-Dihydro-4,6-diphenylpyrimidin-2(1H)-ones

A mixture of acetophenone (1.0 mmol), aldehyde (1.0 mmol), urea (1.5 mmol) and Amberlyst 15 DRY (50 mg) in anhydrous ethanol (10 mL) was refluxed for an appropriate time as indicated by TLC. After completion of the reaction the catalyst was filtered and washed with ethyl acetate until free from organic material. The solvent was evaporated at reduced pressure and the solid obtained was recrystallised from ethanol to afford pure 3,4-dihydro-4,6-diphenylpyrimidine-2(1*H*)-ones **2**(**a–f**) in excellent yields.

### 3.4. General Procedure for the Reaction of Cyclohexanone, Aldehydes, and Urea

A mixture of cyclohexanone (1.0 mmol), aldehyde (2.0 mmol), urea (3.0 mmol) and Amberlyst 15 DRY (50 mg) in anhydrous ethanol (10 mL) was refluxed for an appropriate time as indicated by TLC. After completion of the reaction the catalyst was filtered and washed with ethyl acetate until free from organic material. The solvent was evaporated at reduced pressure and the solid obtained was recrystallised from ethanol to afford the desired spirofused heterotricyclic products **3**(**a–d**) in 85–92% yield.

### 3.5. Spectral Data of Compounds

**5-(Ethoxycarbonyl)-6-methyl-4-phenyl-3,4-dihydropyrimidin-2(1H)-one (1a)**

Mp 205–207°C; ^1^HNMR (DMSO-*d*_6_) *δ*: 1.09 (t, 3H, *J* = 7.1 Hz, OCH_2_CH_3_), 2.25 (s, 3H, CH_3_), 3.97 (q, 2H, *J* = 7.1 Hz, OCH2), 5.05 (d, 1H, *J* = 2.15 –CH), 7.28 (m, 5H, Ar-H), 7.75 (s, 1H, NH), 9.20 (s, 1H, NH); ^13^C-NMR (DMSO-*d*_6_) *δ*: 14.11, 17.94, 54.91, 60.05, 100.95, 112.85, 113.05, 125.15, 125.81, 129.05, 131.20, 150.16, 155.47, 163.81; IR (*ν*_max⁡._; KBr, cm^−1^): 3240, 1722, 1638; ESI-MS 261 (M + H); C_14_H_16_N_2_O_3_ (260.29); Calcd. C, 64.60; H, 6.20; N, 10.76; O, 18.44. Found. C, 64.63; H, 6.18; N, 10.73; O, 18.47.

**5-(Ethoxycarbonyl)-4-(4-methoxyphenyl)-6-methyl-3,4-dihydropyrimidin-2(1H)-one (1b)**

Mp 202-203°C; ^1^H-NMR (DMSO-*d*_6_) *δ*: 1.15 (t, 3H, *J* = 7.12 Hz, OCH_2_CH_3_), 2.33 (s, 3H, CH_3_), 3.78 (s, 3H, –OCH_3_), 4.06 (q, 2H, *J* = 7.12 Hz, OCH_2_CH_3_), 5.34 (d, 1H, *J* = 2.28 –CH), 6.82 (d, 2H, *J* = 8.60, Ar-H), 7.22 (d, 2H, *J* = 8.60, Ar-H), 7.76 (s, 1H, NH), 9.26 (s, 1H, NH); ^13^C-NMR (DMSO-*d*_6_) *δ*:14.32, 18.80, 55.23, 55.40, 60.17, 101.68, 114.06, 127.97, 136.22, 146.16, 153.59, 159.30, 165.87; IR (*ν*_max⁡._; KBr, cm^−1^): 3232, 1720, 1638; ESI-MS 291 (M + H); C_15_H_18_N_2_O_4_ (290.31); Cacld. C, 62.06; H, 6.25; N, 9.65; O, 22.04. Found. C, 62.08; H, 6.22; N, 9.69; O, 22.02.

**5-(Ethoxycarbonyl)-4-(4-dimethylamino-phenyl)-6-methyl-3,4-dihydropyrimidin-2(1H)-one (1c)**

Mp 254–256°C; ^1^H-NMR (DMSO-*d*_6_) *δ*: 0.99 (t, 3H, *J* = 7.12 Hz, OCH_2_CH_3_), 2.11 (s, 3H, CH_3_), 2.84 (s, 6H, N(CH_3_)_2_), 4.09(q, 2H, *J* = 7.12 Hz, OCH_2_CH_3_), 5.05 (d, 1H, *J* = 2.21, –CH), 6.42 (d, 2H, *J* = 8.55, Ar-H), 7.12 (d, 2H, *J* = 8.56, Ar-H), 7.15 (s, 1H, NH), 9.05 (s, 1H, NH); ^13^C-NMR (DMSO-*d*_6_) *δ*: 14.28, 18.78, 44.47, 55.23, 60.15, 101.60, 112.05, 125.65, 134.25, 141.16, 153.46, 159.02, 165.24; IR (*ν*_max⁡._; KBr, cm^−1^): 3242, 1721, 1637; ESI-MS 304 (M + H); C_16_H_21_N_3_O_3_; (303.36); Calcd. C, 63.35; H, 6.98; N, 13.85; O, 15.82. Found. C, 63.38; H, 6.93; N, 13.87; O, 15.79.

**5-(Ethoxycarbonyl)-4-(4-nitrophenyl)-6-methyl-3,4-dihydropyrimidin-2(1H)-one (1d)**

Mp 212-213°C; ^1^H-NMR (DMSO-*d*_6_) *δ*: 1.11 (t, 3H, *J* = 7.04 Hz, OCH_2_CH_3_), 2.32 (s, 3H, CH_3_), 4.03 (q, 2H, *J* = 7.12 Hz, OCH_2_CH_3_), 5.78 (d, 1H, *J* = 2.28, –CH), 7.51 (d, 2H, *J* = 9.18, Ar-H), 7.69 (s, 1H, NH), 8.16 (d, 2H, *J* = 9.16, Ar-H), 9.05 (s, 1H, NH); ^13^C-NMR (DMSO-*d*_6_) *δ*: 14.22, 18.71, 55.81, 60.15, 101.60, 118.15, 130.37, 138.34, 152.26, 153.41, 159.15, 165.85; IR (*ν*_max⁡._; KBr, cm^−1^): 3235, 1740, 1631; ESI-MS 306 (M + H); C_14_H_15_N_3_O_5_; (305.29); Calcd. C, 55.08; H, 4.95; N, 13.76; O, 26.20. Found. C, 55.10; H, 4.93; N, 13.79; O, 26.16.

**5-(Ethoxycarbonyl)-4-(4-chlorophenyl)-6-methyl-3,4-dihydropyrimidin-2(1H)-one (1e)**

Mp 214-215°C; ^1^H-NMR (DMSO-*d*_6_) *δ*: 1.12 (t, 3H, *J* = 7.14 Hz, OCH_2_CH_3_), 2.30 (s, 3H, CH_3_), 3.91 (q, 2H, *J* = 7.16 Hz, OCH_2_CH_3_), 5.70 (d, 1H, *J* = 2.28, –CH), 7.21 (d, 2H, *J* = 9.18, Ar-H), 7.69 (s, 1H, NH), 7.94 (d, 2H, *J* = 9.18, Ar-H), 9.16 (s, 1H, NH); ^13^C-NMR (DMSO-*d*_6_) *δ*: 14.18, 18.62, 55.72, 60.21, 101.55, 118.17, 130.32, 142.29, 152.31, 153.39, 159.17, 165.83; IR (*ν*_max⁡._; KBr, cm^−1^): 3225, 1720, 1615; ESI-MS 295 (M + H); C_14_H_15_ClN_2_O_3_; Calcd. C, 57.05; H, 5.13; Cl, 12.03; N, 9.50; O, 16.29. Found. C, 57.08; H, 5.10; Cl, 12.06; N, 9.47; O, 16.31.

**5-(Methoxycarbonyl)-4-(4-nitrophenyl)-6-methyl-3,4-dihydropyrimidin-2(1H)-one (1f)**

Mp 237–239°C; ^1^H-NMR (DMSO-*d*_6_) *δ*: 2.21 (s, 3H, CH_3_), 3.90 (s, 3H, –COOCH_3_), 5.51 (d, 1H, *J* = 2.15, –CH), 7.42 (d, 2H, *J* = 9.11, Ar-H), 7.44 (s, 1H, NH), 8.05 (d, 2H, *J* = 9.10, Ar-H), 9.05 (s, 1H, NH); ^13^C-NMR (DMSO-*d*_6_) *δ*: 18.64, 52.40, 55.40, 109.60, 113.23, 128.31, 137.20, 149.65, 155.45, 160.36, 166.20; IR (*ν*_max⁡._; KBr, cm^−1^): 3232, 1724, 1631; ESI-MS 292 (M + H); C_13_H_13_N_3_O_5_; (291.26); Calcd. C, 53.61; H, 4.50; N, 14.43; O, 27.47. Found. C, 53.64; H, 4.47, N, 14.46; O, 27.44.

**5-(Methoxycarbonyl)-4-(4-methoxyphenyl)-6-methyl-3,4-dihydropyrimidin-2(1H)-one (1g)**

Mp 192-193°C; ^1^H-NMR (DMSO-*d*_6_) *δ*: 2.24 (s, 3H, CH_3_), 3.92 (s, 3H, –COOCH_3_), 3.75 (s, 3H, –OCH_3_), 5.22 (d, 1H, *J* = 2.21 –CH), 6.76 (d, 2H, *J* = 8.58, Ar-H), 7.18 (d, 2H, *J* = 8.58, Ar-H), 7.62 (s, 1H, NH), 9.15 (s, 1H, NH); ^13^C-NMR (DMSO-*d*_6_) *δ*: 18.61, 53.36, 55.05, 55.87, 108.54, 113.21, 128.47, 137.64, 148.54, 154.16, 160.81, 165.94; IR (*ν*_max⁡._; KBr, cm^−1^): 3242, 1721, 1637; ESI-MS 277 (M + H); C_14_H_16_N_2_O_4_; (276.29); Calcd. C, 60.86; H, 5.84; N, 10.14; O, 23.16. Found. C, 60.89; H, 5.81, N, 10.17; O, 23.13.

**5-(Ethoxycarbonyl)-6-methyl-4-styryl-3,4-dihydropyrimidin-2(1H)-one (1h)**

Mp 231–233°C; ^1^H-NMR (DMSO-*d*_6_) *δ*: 1.20 (t, 3H, *J* = 7.0 Hz, OCH_2_CH_3_), 2.21 (s, 3H, CH_3_), 4.09 (q, 2H, *J* = 7.05 Hz, OCH_2_CH_3_), 4.74 (d, 1H, *J* = 4.80, –CH), 6.20 (dd, *J* = 15.8, 6.0 Hz, 1H, CH=C–H), 6.37 (d, *J* = 15.9 Hz, 1H, H–C=CH) 7.21–7.46 (m, 5H, Ar-H), 7.53 (s, 1H, NH), 9.14 (s, 1H, NH); ^13^C-NMR (DMSO-*d*_6_) *δ*: 14.21, 17.31, 51.84, 59.45, 98.54, 127.34, 128.54, 129.54, 130.59, 131.24, 135.24, 145.34, 153.62, 165.23; IR (*ν*_max⁡._; KBr, cm^−1^): 3242, 1704, 1652; ESI-MS 287 (M + H); C_16_H_18_N_2_O_3_; (286.33); Calcd. C, 67.12; H, 6.34; N, 9.78; O, 16.76. Found. C, 67.15; H, 6.32; N, 9.81; O, 16.73.

**5-(Ethoxycarbonyl)-4-(3-nitrophenyl)-6-methyl-3,4-dihydropyrimidin-2(1H)-thione (1i)**

Mp 206-207°C; ^1^H-NMR (DMSO-*d*_6_) *δ*: 1.15 (t, 3H, *J* = 7.14 Hz, OCH_2_CH_3_), 2.27 (s, 3H, CH_3_), 4.02 (q, 2H, *J* = 7.11 Hz, OCH_2_CH_3_), 5.81 (d, 1H, *J* = 2.06, –CH), 7.23–7.37 (m, 4H, Ar-H), 7.78 (s, 1H, NH), 9.34 (s, 1H, NH); ^13^C-NMR (DMSO-*d*_6_) *δ*: 14.14, 18.60, 55.64, 60.21, 101.34, 126.25, 128.02, 129.32, 130.75, 135.65, 144.34, 160.40, 165.64, 182.65; IR (*ν*_max⁡._; KBr, cm^−1^): 3245, 1725, 1632, 1575, 1545; ESI-MS 322 (M + H); C_14_H_15_N_3_O_4_S; (321.35); Calcd. C, 52.33; H, 4.70; N, 13.08; O, 19.92; S, 9.98. Found. C, 52.36; H, 4.66; N, 13.11; O, 19.88; S, 9.99.

**5-(Ethoxycarbonyl)-4-(4-methoxyphenyl)-6-methyl-3,4-dihydropyrimidin-2(1H)-thione (1j)**

Mp 154–156°C; ^1^H-NMR (DMSO-*d*_6_) *δ*: 1.17 (t, 3H, *J* = 7.11 Hz, OCH_2_CH_3_), 2.37 (s, 3H, CH_3_), 4.12 (s, 3H, –OCH_3_), 4.15 (q, 2H, *J* = 7.10 Hz, OCH_2_CH_3_), 5.44 (d, 1H, *J* = 2.15 –CH), 7.11 (d, 2H, *J* = 8.15, Ar-H), 7.37 (d, 2H, *J* = 8.11, Ar-H), 7.84 (s, 1H, NH), 9.43 (s, 1H, NH); ^13^C-NMR (DMSO-*d*_6_) *δ*: 14.32, 18.05, 55.24, 55.49, 60.45, 101.84, 114.32, 127.74, 137.25, 147.15, 159.45, 165.62, 182.48; IR (*ν*_max⁡._; KBr, cm^−1^): 3240, 1725, 1635, 1574, 1540; ESI-MS 307 (M + H); C_15_H_18_N_2_O_3_S; (306.38); Calcd. C, 58.80; H, 5.92; N, 9.14; O, 15.67; S, 10.47. Found. C, 58.84; H, 5.89; N, 9.17; O, 15. 64; S, 10.49.

**5-(Ethoxycarbonyl)-4-(3-1H-indole)-6-methyl-3,4-dihydropyrimidin-2(1H)-one (1n)**

Mp. 212–214°C; ^1^H-NMR (DMSO-*d*_6_) *δ*: 9.17 (s, 1H, NH), 7.04 (s, 1H, NH), 8.48 (s, 1H, NH), 7.76 (s, 1H), 7.18–7.34 (m, 4H), 5.23 (d, 1H, *J* = 3.7 Hz), 3.97 (q, 2H, *J* = 7.2 Hz), 2.24 (s, 3H), 1.15 (t, 3H, *J* = 7.2 Hz); ^13^C-NMR (DMSO-*d*_6_) *δ*: 172.10, 155.25, 152.90, 136.90, 127.30, 123.20, 121.80, 119.10, 118.90, 111.15, 106.90, 104.35, 60.10, 34.15, 14.90, 13.90. IR (*ν*_max⁡._; KBr, cm^−1^): 3417, 3356, 3240, 2978, 1702, 1653, 1538, 1187, 1085, 870; ESI-MS 300 (M + H); C_16_H_17_N_3_O_3_; (299.32); Calcd: C, 64.20; H, 5.72; N, 14.04; O, 16.04. Found: C, 63.89; H, 5.93; N, 14.37; O, 16.09.

**5-(Ethoxycarbonyl)-4-(3-quinoline)-6-methyl-3,4-dihydropyrimidin-2(1H)-one (1q)**

Mp 245–247°C; ^1^H-NMR (DMSO-*d*_6_) *δ*: 9.25 (s, 1H, NH), 7.73 (s, 1H, NH), 8.32 (s, 1H), 7.63–7.79 (m, 4H), 7.80 (s, 1H), 5.12 (d, 1H, *J* = 2.8 Hz), 4.11 (q, 2H, *J* = 7.5 Hz), 2.28 (s, 3H), 1.09 (t, 3H, *J* = 7.5 Hz); ^13^C-NMR (DMSO-*d*_6_) *δ*: 172.50, 155.25, 153.35, 148.10, 147.15, 135.05, 135.05, 129.10, 127.30, 126.45, 126.10, 104.50, 60.10, 53.00, 14.90, 13.90; IR (*ν*_max⁡._; KBr, cm^−1^): 3408, 3365, 3280, 1698, 1640, 1513, 1227, 779; ESI-MS 312 (M + H); C_17_H_17_N_3_O_3_ (311.34); Calcd: C, 65.58; H, 5.50; N, 13.50; O, 15.42. Found: C, 65.63; H, 5.61; N, 13.42; O, 15.37.

**5-(Ethoxycarbonyl)-4-(2-pyrimidine)-6-methyl-3,4-dihydropyrimidin-2(1H)-one (1r)**

Mp 255–257°C; ^1^H-NMR (DMSO-*d*_6_) *δ*: 9.20 (s, 1H, NH), 7.65 (s, 1H, NH), 8.42 (d, 2H, *J* = 7.5 Hz), 7.38 (t, 1H, *J* = 7.5 Hz), 5.10 (d, 1H, *J* = 3.5 Hz), 4.02 (q, 2H, *J* = 7.0 Hz), 2.23 (s, 3H), 1.11 (t, 3H, *J* = 7.0 Hz); ^13^C-NMR (DMSO-*d*_6_) *δ*: 172.60, 168.70, 157.20, 155.85, 153.85, 119.90, 104.20, 60.10, 56.10, 15.15, 13.90; IR (*ν*_max⁡._; KBr, cm^−1^): 3413, 3385, 3245, 2965, 1709, 1658, 1540, 1235, 1090, 780; ESI-MS 263 (M + H); C_12_H_14_N_4_O_3_; (262.26); Calcd: C, 54.96; H, 5.38; N, 21.36; O, 18.30. Found: C, 54.84; H, 5.29; N, 22.04; O, 18.75.

**5-(Ethoxycarbonyl)-4-(4-hydroxyl-2H(1)-benzopyran-2-one-3-yl)-6-methyl-3,4-dihydropyrimi din-2(1H)-one (1t)**

277–279°C; ^1^H-NMR (DMSO-*d*_6_) *δ*: 11.85 (s, 1H, OH), 9.80 (s, 1H, NH), 7.69 (s, 1H, NH), 7.30–7.80 (m, 4H), 4.85 (d, 1H, *J* = 3.5 Hz), 4.23 (q, 2H, *J* = 6.8 Hz), 2.35 (s, 3H), 1.21 (t, 3H, *J* = 6.8 Hz); ^13^C-NMR (DMSO-*d*_6_) *δ*: 172.50, 171.20, 164.15, 155.10, 153.60, 152.20, 131.15, 129.80, 122.30, 121.10, 117.80, 116.25, 94.50, 60.20, 43.25, 15.15, 14.15; IR (*ν*_max⁡._; KBr, cm^−1^): 3389, 3240, 2943, 1721, 1705, 1619, 1562, 1235, 1123, 810; ESI-MS 345 (M + H); C_17_H_16_N_2_O_6_; (344.32); Calcd: C, 59.30; H, 4.68; N, 8.14; O, 27.88. Found: C, 59.24; H, 4.76; N, 8.07; O, 28.01.

**4,6-diphenyl-3,4-dihydropyrimidin-2(1H)-one (2a)**

Mp 233–236°C; ^1^H-NMR (DMSO-*d*_6_) *δ*: 9.51 (s, 1H, NH), 9.21 (s, 1H, NH), 7.21–7.62 (m, 10H, Ar-H), 5.20 (d, 1H, *J* = 4.1 Hz, C=CH), 5.12 (d, 1H, *J* = 4.1 Hz, CH); ^13^C-NMR (DMSO-*d*_6_) *δ*: 150.2, 143.2, 136.6, 134.2, 128.6, 126.4, 128.7, 126.9, 97.5, 51.9; IR (*ν*_max⁡._; KBr, cm^−1^): 3312, 1685, 1598, 1449; ESI-MS 251 (M + H); C_16_H_14_N_2_O; (250.30); Calcd. C, 76.78; H, 5.64; N, 11.19; O, 6.39. Found. C, 76.53; H, 5.34; N, 11.02; O, 6.13.

**4-(4-chlorophenyl)-6-phenyl-3,4-dihydropyrimidin-2(1H)-one (2b)**

Mp 267–269°C; ^1^H-NMR (DMSO-*d*_6_) *δ*: 9.42 (s, 1H, NH), 9.12 (s, 1H, NH), 7.19–7.78 (m, 9H, Ar-H), 5.60 (d, 1H, *J* = 4.3 Hz, C=CH), 5.01 (d,1H, *J* = 4.3 Hz, CH); ^13^C-NMR (DMSO-*d*_6_) *δ*: 150.2, 141.3, 136.6, 134.2, 132.3, 128.6, 128.3, 128.7, 126.4, 97.5, 51.9; IR (*ν*_max⁡._; KBr, cm^−1^): 3319, 1683, 1569, 1463; ESI-MS 285 (M + H); C_16_H_13_ClN_2_O_3_; (284.74); Calcd. C, 67.49; H, 4.60; Cl, 12.45, N, 9.84, O, 5.62. Found. C, 67.18; H, 4.24; Cl, 12.21; N, 9.32, O, 5.41.

**4-(4-methoxyphenyl)-6-phenyl-3,4-dihydropyrimidin-2(1H)-one (2d)**

Mp 259–261°C; ^1^H-NMR (DMSO-*d*_6_) *δ*: 9.23 (s, 1H, NH), 8.87 (s, 1H, NH), 7.18–7.56 (m, 9H, Ar-H), 5.85 (d, 1H, *J* = 5.6 Hz, C=CH), 5.26 (d, 1H, *J* = 5.6 Hz, CH), 3.69 (s, 3H, OCH_3_); ^13^C-NMR (DMSO-*d*_6_) *δ*: 158.6, 150.2, 136.6, 135.5, 134.2, 127.9, 114.1, 128.7, 128.0, 126.4, 97.5, 55.8, 51.9; IR (*ν*_max⁡._; KBr, cm^−1^): 3345, 1645, 1536, 1422; ESI-MS 281 (M + H); C_17_H_16_N_2_O_2_; (280.32); Calcd. C, 72.84; H, 5.75, N, 9.99; O, 11.42. Found. C, 72.53; H, 5.42; N, 9.73; O, 11.19.

**4,8-diphenyloctahydro-1H-pyrimido[5,4-i]quinazoline-2,10(3H,11H)-dione (3a)**

Mp 327–329°C; ^1^H-NMR (DMSO-*d*_6_) *δ*: 7.40–7.19 (m, 10 H), 7.08 (s, 1H), 6.97 (s, 1H), 6.62 (s, 1H), 6.39 (s, 1H), 4.50 (d, 1H), 4.82 (d, 1H), 2.02 (m, 2H), 1.38 (m, 2H), 1.24 (m, 2H), 0.82 (t, 2H); ^13^C-NMR (DMSO-*d*_6_) *δ*: 155.9, 140.5, 128.1, 128.6, 126.0, 63.7, 50.2, 49.1, 17.8; ESI-MS 377 (M + H); C_22_H_24_N_4_O_2_; (376.45); Calcd. C, 70.19; H, 6.43; N, 14.88; O, 8.50. Found. C, 70.03; H, 6.21; N, 14.45; O, 8.23.

**4,8-bis(2-chlorophenyl)octahydro-1H-pyrimido[5,4-i]quinazoline-2,10(3H, 11H)-dione (3d)**

Mp 321–323°C; ^1^H-NMR (DMSO-*d*_6_) *δ*: 7.42 (s, 1H), 7.35–7.10 (m, 9H), 6.75 (s, 1H), 5.32 (s, 1H), 5.32 (s, 1H), 3.91 (m, 3H), 3.69 (m, 3H), 2.30 (m, 2H), 2.01 (m, 1H), 1.84 (m, 1H), 1.32 (m, 1H), 1.19 (m, 1H), 0.89 (m, 1H); ^13^C-NMR (DMSO-*d*_6_) *δ*: 155.9, 140.5, 133.4, 129.5, 128.6, 127.4, 63.7, 48.6, 45.1, 23.6, 17.8; ESI-MS 445 (M + H); C_22_H_22_Cl_2_N_4_O_2_ (445.34); Calcd. C, 59.33; H, 4.98; Cl, 15.92; N, 12.58; O, 7.19. Found. C, 59.12; H, 4.56; Cl, 15.74; N, 12.28; O, 7.02.

## 4. Conclusion

In conclusion, we have developed a simple, efficient, environmentally benign, and improved protocol for the synthesis of 3,4-dihydropyrimidin-2(1*H*)-ones/thiones over Amberlyst 15 DRY as the catalyst with excellent yields. The simplicity of the system, ease of separation/reuse of the catalyst due to its heterogeneous nature, excellent yields of the products, and ease of workup fulfill the triple bottom line philosophy of green chemistry and make the present methodology environmentally benign.

## Figures and Tables

**Table 1:** Catalytic activity of different ion exchange resins in Biginelli condensation^a^.

Entry	Ion exchange resin	Reaction time (h)	Yield (%)
1	—	10	Trace
2	Amberlyst-70	3	81
3	Amberlyst 15 DRY^a^	5.5	94
4	Indion-130	3	92.5
5	Indion-190	3.5	92
6	Nafion-H	4.5	85
7	Envirocat EPZ10	6	84
8	Montmorillonite KSF	10	38

^a^Reaction conditions: ethyl acetoacetate (1.0 mmol), benzaldehyde (1.0 mmol), and urea (1.2 mmol) in dry ethanol (10 mL), ion exchange resin (50 mg) at refluxing temperature.

**Table 2:** Optimization of the reaction conditions for the synthesis of **1a** ^a^.

Entry	Catalyst	Solvent	Time (h)	Yield (%)
1	Amberlyst 15 DRY	Water	4	90
2	Amberlyst 15 DRY	EtOH	5.5	94
3	Amberlyst 15 DRY	CH_3_CN	6.5	85
4	Amberlyst 15 DRY	THF	6	87
5	Amberlyst 15 DRY	Benzene	10	Trace
6	Amberlyst 15 DRY	Toluene	10	Trace

^a^All reactions were conducted at reflux temperature of the solvent used.

**Table 3:** Amberlyst 15 DRY catalyzed synthesis of dihydropyrimidine-2-(1*H*)-ones/thiones.

Entry	R^1^	R^2^	X	Products^a^	Yield^b^ (%)	M.P (°C)
1	C_6_H_5_	Et	O	**1a**	89	205–207
2	4-(CH_3_O)–C_6_H_4_	Et	O	**1b**	90	202-203
3	4-(NMe_2_)–C_6_H_4_	Et	O	**1c**	83	254–256
4	4-NO_2_–C_6_H_4_	Et	O	**1d**	94	212-213
5	4-(Cl)–C_6_H_4_	Et	O	**1e**	91	214–215
6	4-(NO_2_)C_6_H_4_	Me	O	**1f**	95	237–239
7	4-(CH_3_O)–C_6_H_4_	Me	O	**1g**	84	192-193
8	C_6_H_5_–CH=CH	Et	O	**1h**	94	231–233
9	3-NO_2_–C_6_H_4_	Et	S	**1i**	92	206-207
10	4-(CH_3_O)–C_6_H_4_	Et	S	**1j**	90	154–156
11	C_4_H_4_N	Et	O	**1k**	83	180–182
12	C_4_H_3_O	Et	O	**1l**	85	211–213
13	C_4_H_3_S	Et	O	**1m**	86	201–203
14	C_8_H_6_N	Et	O	**1n**	80	212–214
15	C_5_H_4_N	Et	O	**1o**	91	193-194
16	C_5_H_4_N	Et	S	**1p**	85	168-169
17	C_9_H_6_N	Et	O	**1q**	91	245–247
18	C_4_H_3_N_2_	Et	O	**1r**	89	255–257
19	C_10_H_7_	Et	O	**1s**	92	182–185
20	C_9_H_5_O_3_	Et	O	**1t**	89	277–279

^a^Reaction conditions: *β*-ketoester (1.0 mmol), aldehyde (1.0 mmol), and urea/thiourea (1.2 mmol) in dry ethanol (10 mL), ion exchange resin (50 mg) at refluxing temperature. ^b^Isolated yields.

**Table 4:** Amberlyst 15 DRY catalyzed synthesis of 5-unsubstitued 3,4-dihydropyrimidin-2(1*H*)-ones **2**(**a**–**f**).

Entry	R	Products^a^	Yield^b^ (%)	M.P (°C)
1	C_6_H_5_	**2a**	90	233–236
2	4-(Cl)–C_6_H_4_	**2b**	92	267–269
3	4-(CH_3_)–C_6_H_4_	**2c**	86	248–250
4	4-(CH_3_O)–C_6_H_4_	**2d**	84	259–261
5	2-(Cl)–C_6_H_4_	**2e**	91	260–263
6	3-(CH_3_O) –C_6_H_4_	**2f**	88	256–258

^a^Reaction conditions: acetophenone (1.0 mmol), benzaldehyde (1.0 mmol), and urea (1.5 mmol) in dry ethanol (10 mL), ion exchange resin (50 mg) at refluxing temperature. ^b^Isolated yields.

**Table 5:** Amberlyst 15 DRY catalyzed reaction of cyclohexanone, aldehyde, and urea.

Entry	R	Products^a^	Yield^b^ (%)	M.P (°C)
1	C_6_H_5_	**3a**	93	327–329
2	4-(NO_2_)C_6_H_4_	**3b**	85	341–343
3	4-(CH_3_)C_6_H_4_	**3c**	89	348–351
4	2-(Cl)–C_6_H_4_	**3d**	88	321–323

^a^Reaction conditions: cyclohexanone (1.0 mmol), aldehyde (2.0 mmol), and urea (3.0 mmol) in dry ethanol (10 mL), ion exchange resin (50 mg) at refluxing temperature. ^b^Isolated yields.

**Table 6:** Physical properties of Amberlyst 15 DRY.

Physical form	Opaque beads
Ionic form as shipped	Hydrogen
Concentration of acid sites	≥4.7 eq/Kg
Water content	≤1.5% (H^+^ form)
Shipped weight	610 g/L (38 lbs/ft)
Fines content	<0.300 mm: 1.0% max
Surface area	45 m^2^/g
Average pore diameter	250 Å
Swelling	
60 to 70% (dry to Water)	
10 to 15% (dry to hexane)	
10 to 15% (dry to toluene)	
15 to 20% (dry to ethylene dichloride)	
30 to 40% (dry to ethyl acetate)	
60 to 70% (dry to ethyl alcohol, 95%)	
15 to 20% (dry to phenol)	
3 to 5% (dry to benzene)	
